# Estimation of current and post-treatment retinal function in chronic central serous chorioretinopathy using artificial intelligence

**DOI:** 10.1038/s41598-021-99977-4

**Published:** 2021-10-14

**Authors:** Maximilian Pfau, Elon H. C. van Dijk, Thomas J. van Rijssen, Steffen Schmitz-Valckenberg, Frank G. Holz, Monika Fleckenstein, Camiel J. F. Boon

**Affiliations:** 1 Department of Ophthalmology, University of Bonn, Bonn, Germany; 2 National Eye Institute, National Institutes of Health, Bethesda, MD USA; 3 Department of Ophthalmology, Leiden University Medical Center, P. O. Box 9600, 2300 RC Leiden, The Netherlands; 4 John A. Moran Eye Center, University of Utah, Utah, USA; 5 Department of Ophthalmology, Amsterdam University Medical Centers, University of Amsterdam, Amsterdam, The Netherlands

**Keywords:** Predictive markers, Outcomes research, Eye manifestations

## Abstract

Refined understanding of the association of retinal microstructure with current and future (post-treatment) function in chronic central serous chorioretinopathy (cCSC) may help to identify patients that would benefit most from treatment. In this post-hoc analysis of data from the prospective, randomized PLACE trial (NCT01797861), we aimed to determine the accuracy of AI-based inference of retinal function from retinal morphology in cCSC. Longitudinal spectral-domain optical coherence tomography (SD-OCT) data from 57 eyes of 57 patients from baseline, week 6–8 and month 7–8 post-treatment were segmented using deep-learning software. Fundus-controlled perimetry data were aligned to the SD-OCT data to extract layer thickness and reflectivity values for each test point. Point-wise retinal sensitivity could be inferred with a (leave-one-out) cross-validated mean absolute error (MAE) [95% CI] of 2.93 dB [2.40–3.46] (scenario 1) using random forest regression. With addition of patient-specific baseline data (scenario 2), retinal sensitivity at remaining follow-up visits was estimated even more accurately with a MAE of 1.07 dB [1.06–1.08]. In scenario 3, month 7–8 post-treatment retinal sensitivity was predicted from baseline SD-OCT data with a MAE of 3.38 dB [2.82–3.94]. Our study shows that localized retinal sensitivity can be inferred from retinal structure in cCSC using machine-learning. Especially, prediction of month 7–8 post-treatment sensitivity with consideration of the treatment as explanatory variable constitutes an important step toward personalized treatment decisions in cCSC.

## Introduction

Chronic central serous chorioretinopathy (CSC) is a relatively common chorioretinal disease, associated with a loss of central vision due to the accumulation of subretinal fluid (SRF)^1,2^. While the exact underlying molecular pathology of CSC remains uncertain, it has been established that CSC predominantly affects middle-aged men and may be associated with corticosteroid use, stress, endocrine diseases, and genetic susceptibility factors^1–3^. Despite its relatively high prevalence, treatment remains controversial. However, some degree of evidence-based consensus for the treatment of chronic CSC (cCSC) has emerged recently^1^.

Two recent randomized clinical trials and a range of large retrospective studies have highlighted the superiority of photodynamic therapy (PDT) for the treatment of cCSC, as compared to the outcomes of micropulse laser treatment, oral mineralocorticoid antagonist treatment (e.g. eplerenone), or intravitreal anti-vascular endothelial growth factor treatment^1,4,5^. The PLACE trial was the first large prospective interventional trial in cCSC, in which half-dose PDT has been found to be superior to high-density subthreshold micropulse laser (HSML) treatment, both in terms of anatomical and functional outcome measures. Despite the highly significant difference in SRF resolution rate on spectral-domain optical coherence tomography (SD-OCT) in favor of half-dose PDT, no significant difference in the best-corrected visual acuity improvement was observed between the 2 interventions^4^. In contrast, fundus-controlled perimetry (FCP, also termed *“microperimetry”*) revealed significant between-group differences for the change in mesopic retinal sensitivity. Yet, the statistical effect size was smaller compared to anatomical differences^4^, which is most likely attributable to the retest-variability of FCP testing^6^. Moreover, the maximum attainable spatial resolution and retinal coverage of FCP testing is limited due to test time, patient fatigue, and psychophysical factors (increase in retest variability for smaller stimuli)^7^.

Recently, the idea of applying supervised machine-learning to infer retinal function from SD-OCT has been brought forward by multiple groups in the setting of macular telangiectasia type 2^8^, choroidal neovascularization and geographic atrophy secondary to age-related macular degeneration (AMD)^9,10^, as well as Leber congenital amaurosis (LCA)^11^. This strategy potentially allows to obtain a close surrogate of function—even in patients unfit for psychophysical testing—using ubiquitously available SD-OCT imaging. Based on the size of the scan field covered by SD-OCT, functional maps of the central macula can be obtained^8–10^. Previously, we have introduced the term “inferred sensitivity’” maps for this approach^9,10^.

Prior to the application of any predictive models including mapping of inferred sensitivity, a disease-specific validation of the prediction accuracy is necessary, as the feature importance likely varies among different diseases. Besides the inference of sensitivity using SD-OCT data from the same visit, prediction of sensitivity at future visits from baseline SD-OCT data in eyes undergoing therapeutic interventions would be particularly helpful. For example, patients could be informed regarding the expected outcome, which would represent an important step toward personalized medicine^11^.

Accordingly, in this study we aimed to analyze how closely retinal anatomical parameters correlate to retinal sensitivity in cCSC based on data acquired in the PLACE trial^4,12^. Specifically, we aimed (i) to assess the accuracy for inference of retinal sensitivity in an “unknown patient" (i.e., without prior data). In addition, (ii) we evaluated the increase in the prediction accuracy through addition of limited patient-specific functional data (i.e., clinical scenario of FCP testing available at baseline). Finally, (iii) we trained a model to predict month 7–8 post-treatment retinal sensitivity (future outcome) from baseline data aiming to estimate the retinal sensitivity in cCSC patients.

## Results

A total of 57 eyes from 57 cCSC patients (9 female) with a median [IQR] age of 48.79 years [42.80, 52.20] and best-corrected visual acuity of 0.12 LogMAR [0.02, 0.20] at baseline were included in this analysis. Out of these patients, 29 were randomized to half-dose PDT and 28 to HSML (Table 1). The mean retinal sensitivity at baseline was (mean estimate [95% CI]) 21.86 dB [20.80–22.92] and increased (pooled among both treatment groups) with + 1.91 dB [1.67–2.16] at week 6–8 post-treatment and with + 3.13 dB [2.88–3.37] at month 7–8 post-treatment. Based on mixed-model analysis of the point-wise retinal sensitivity data, the differences between baseline and both follow-up visits (baseline to week 6–8 difference: + 1.91 dB, P < 0.001; baseline to month 7–8 difference: + 3.13, P < 0.001) as well as between the 2 follow-up visits (+ 1.21 dB, P < 0.001) were statistically significant.

**Table 1:** Baseline characteristics of chronic central serous chorioretinopathy patients included in the current study.

	Overall cohort	Half-dose photodynamic therapy	High-density subthreshold micropulse laser treatment
N	57	29	28
Sex	9 female, 48 male	6 female, 23 male	3 female, 25 male
Age in years (median [IQR])	48.79 [42.80, 52.20]	49.33 [41.96, 53.06]	48.54 [43.23, 50.64]
Best-corrected visual acuity in LogMAR (median [IQR])	0.12 [0.02, 0.20]	0.14 [0.02, 0.20]	0.11 [0.04, 0.22]

*IQR* interquartile range, *LogMAR* logarithm of the minimum angle of resolution.

### Scenario 1

**Figure 1:**
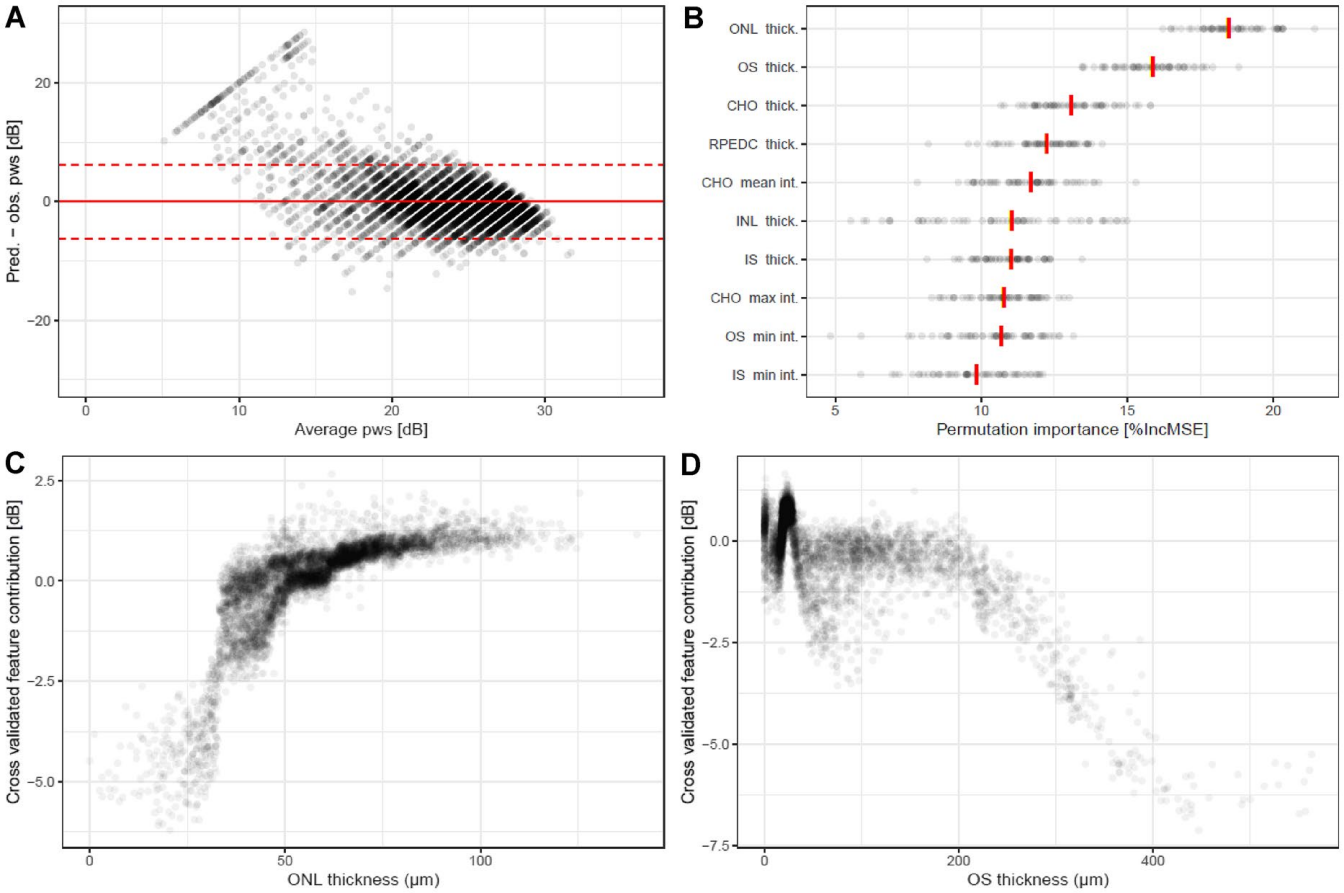
Accuracy of retinal sensitivity predictions for an “unknown patient”. **(A)** Shows a Bland–Altman plot for the point-wise differences between estimated and observed retinal sensitivity. The red solid line shows the mean difference, the red dashed lines the 95% limits of agreement. **(B)** Shows the feature importance in terms of the percentage increase in mean squared error (%IncMSE) for the 10 most relevant features. Each dot denotes the feature importance estimate for a given iteration of the outer cross-validation. Of note, across all iterations of the outer cross-validation, the outer nuclear layer thickness and photoreceptor outer segment thickness constituted the most important imaging feature. The red vertical lines indicate the median feature importance across folds. As shown in the feature contribution plots in **(C,D)**, an outer nuclear layer thickness of 40 µm or less was associated with a marked reduction in retinal sensitivity. Photoreceptor outer segment thickness exhibits a more complex relationship with retinal sensitivity. Outer segment thinning was associated with a reduction in retinal sensitivity, as well as outer segment thickening, which mostly represents subretinal fluid based on the here applied layer definitions. Abbreviations: prediction (pred.), observation (obs.), point-wise retinal sensitivity (pws), thickness (thick.), intensity (int.). Retinal layer: inner nuclear layer (INL), outer nuclear layer (ONL), outer segments (OS), inner segments (IS), retinal pigment epithelium-drusen complex (RPEDC), choroid (CHO).

Without patient-specific functional data and solely based on patient-specific retinal imaging data, the point-wise retinal sensitivity was inferred with a mean absolute error (MAE) of (mean estimate [95% CI]) of 2.93 dB [2.40–3.46], corresponding to a cross-validated R^2^ of 0.513 (Fig. 1A). Examination of the Bland–Altman plots (Fig. 1A) revealed that the predictions were unbiased for sensitivity values between 15 and 36 dB. For loci with more severe loss of function (sensitivity < 15 dB), the predictions overestimated function. The overall estimate of bias [95% CI] was 0.1 dB [− 0.73 to 0.74].

For a new, previously “unknown patient” with cCSC that enters a clinic, a SD-OCT scan would allow to explain about half of the variability in retinal sensitivity without any functional testing. Using the same model and applying it to sequential SD-OCT data, the change in point-wise retinal sensitivity between baseline and week 6–8 post-treatment and between baseline and month 7–8 post-treatment was inferred with a MAE of 3.26 dB [2.66–3.86] (R^2^ of 0.108) and with a MAE of 3.16 dB [2.87–3.44] (R^2^ of 0.200), respectively.

Often, retinal mean sensitivity as a summary metric is evaluated instead of the point-wise sensitivity. Mean sensitivity was inferred with a MAE of 1.96 dB [1.40–2.53] (R^2^ of 0.616). Change in mean sensitivity over time was inferred with a MAE of 1.92 dB [1.24–2.60] (R^2^ of 0.342) for the baseline to week 6–8 post-treatment interval, and with a MAE of 1.66 dB [1.29–2.03] (R^2^ of 0.782) for the baseline to month 7–8 post-treatment interval.

As shown in Fig. 1B, outer nuclear layer (ONL) and photoreceptor outer segments (OS) thickness represented the most important imaging features to infer mesopic retinal sensitivity with a feature importance of (median [IQR]) 18.46 percentage increase in mean squared error (%IncMSE [17.88, 19.45]) and 15.86%IncMSE [14.94, 16.52], respectively. Based on the feature contribution plots, ONL thinning below 50 µm as well as OS compartment thickening, which of note includes SRF, of 200 µm and more were associated with a marked decrease in inferred retinal sensitivity, when present (Fig. 1C,D).

**Figure 2:**
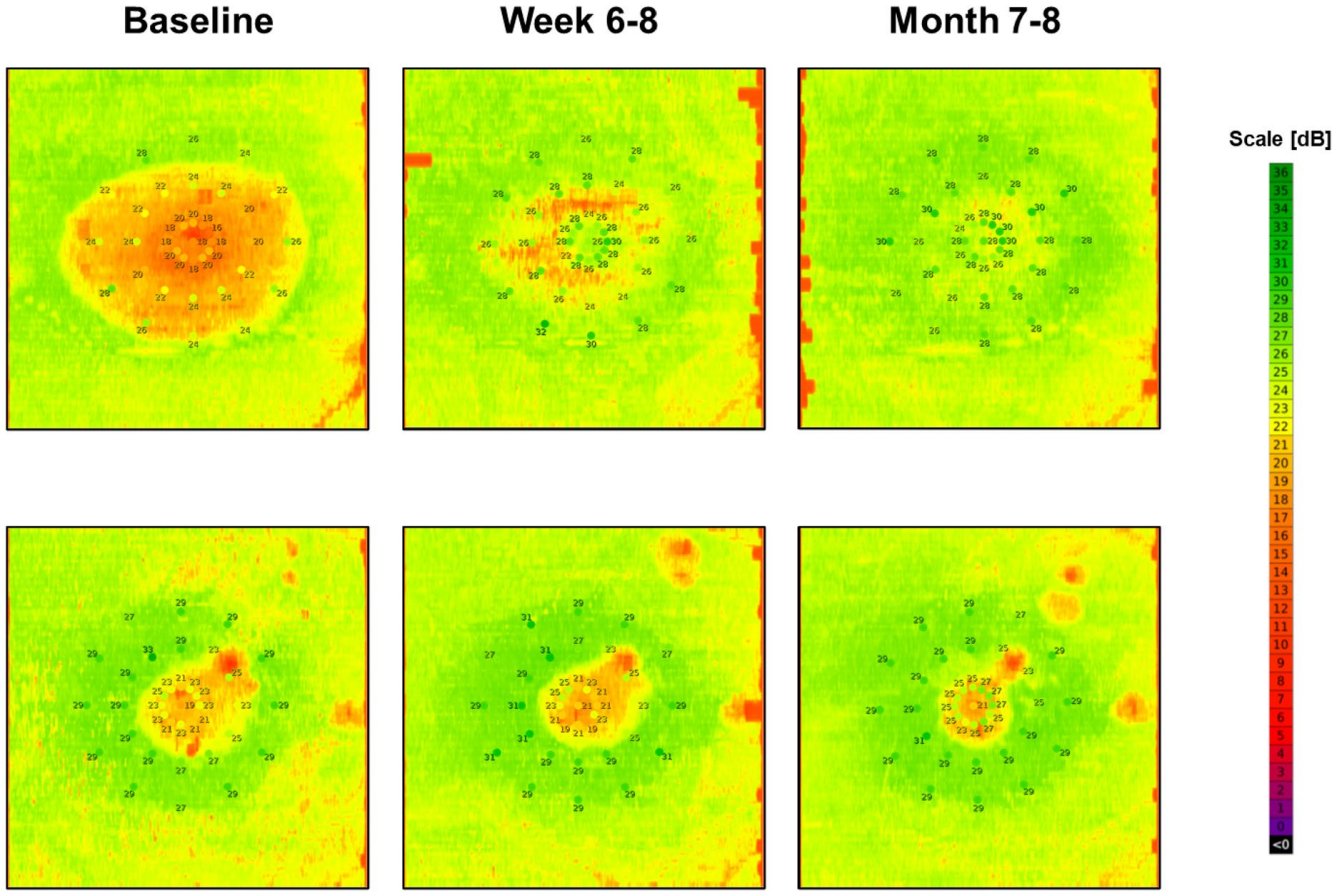
Examples of “inferred sensitivity mapping”. The plots show the cross-validated estimated retinal sensitivity (based on scenario 1) for 3 visits in 2 exemplary patients. The actual fundus-controlled perimetry results are overlayed. The color scale of the device manufacturer was applied for the mapping to facilitate comparisons. Overall, the estimated and observed sensitivity show marked correlation. However, the “inferred sensitivity” maps a superior spatial resolution and coverage of the posterior pole compared to the actual perimetry results. Notably, sensitivity can be estimated for loci between test-points as well as outside of the test pattern. However, the accuracy for predictions outside of the test pattern is unknown.

Figure 2 shows the results of 2 exemplary patients, in whom this model was applied to the complete SD-OCT volume to provide a 2-dimensional map of retinal sensitivity.

### Scenario 2

Patient-specific functional data from the baseline visit was added to the training sets, to evaluate whether this allows to infer retinal sensitivity at the remaining visits even more accurately. Compared to scenario 1, this strategy in scenario 2 markedly lowered both the MAE for point-wise inferred retinal sensitivity (1.07 dB [1.06–1.08] (R^2^ of 0.958)) and the MAE for the mean inferred sensitivity (0.44 dB [0.32–0.56] (R^2^ of 0.972)) (Supplementary Figure S2). Overall, there was no bias in the predictions (+ 0.02 dB [− 0.12 to 0.16]).

Thus, if baseline functional testing and imaging across all visits is available for a given patient, point-wise change in retinal sensitivity may be predicted with a MAE of 0.87 dB [0.70–1.03] (R^2^ of 0.944) for the baseline to week 6–8 post-treatment interval, and with a MAE of 0.87 dB [0.77–0.98] (R^2^ of 0.960) for the baseline to 7–8 months post-treatment interval.

Visual comparison of the patient-wise MAE values between scenario 1 and 2 revealed that addition of patient-specific baseline data improved the prediction accuracy across all patients (Supplementary Figure S2A). In addition, the Bland–Altman plots show that the tendency to overestimate the point-wise retinal sensitivity for test points with low sensitivity in scenario 1 (Supplementary Fig. S2B) was largely reduced through addition of patient-specific training data (Supplementary Figure S2C).

### Scenario 3

Based on one-time imaging at baseline and the type of treatment (half-dose PDT or HSML), the point-wise retinal sensitivity at the final visit (7–8 months post-treatment) was predicted with a MAE of 3.38 dB [2.82–3.94] (R^2^ of 0.249). The corresponding MAE for the mean retinal sensitivity was 2.54 dB [1.93–3.16] (R^2^ of 0.368). The prediction of the future sensitivity did not show bias (− 0.13 dB [− 1.03 to 0.76]).

ONL thickness represented again the most important feature with a (median [IQR]) 14.62%IncMSE [12.91, 16.97]. However, for prediction of retinal sensitivity, the second most important imaging feature was photoreceptor inner segments (IS) thickness (12.37%IncMSE [11.05, 13.47]), followed by retinal pigment epithelium-drusen complex (RPEDC) thickness (11.76%IncMSE [10.30, 13.57]), and the treatment (11.09%IncMSE [8.74, 12.82]). ONL thinning, IS thinning, and RPEDC thickening were associated with loss of function (Supplementary Figure S3C to E). Treatment with half-dose PDT was associated with a markedly higher predicted retinal sensitivity at 7–8 months post-treatment (Supplementary Figure S3F).

## Discussion

The present work evaluated the accuracy of AI-based inference of current and prediction of post-treatment retinal sensitivity from SD-OCT imaging data in patients with cCSC undergoing treatment and followed over a period of 7–8 months. We found a close correlation between structure and function in cCSC at all time points. Inferred sensitivity could potentially substitute or reduce the burden of time-consuming psychophysical testing. The ability to predict post-treatment sensitivity may help to inform patients on their individual prognosis, which is an important step toward personalized medicine.

CSC is a common disease in the working age population, but evidence-based consensus regarding treatment has only recently emerged, mainly based on a range of large randomized treatment trials, which have provided evidence of superiority of half-dose PDT over alternative treatments such as HSML and eplerenone^4,5,19^. With regard to outcome measures, the PLACE trial demonstrated that both anatomical resolution of SRF as well as improvement in mean sensitivity in FCP differed significantly between the 2 arms^4^. However, the P-values for the anatomical outcome measure were much lower compared to measured sensitivity, which may partially be a result of the inherent retest-variability of psychophysical testing. AI-based inference of sensitivity, as previously proposed in macular telangiectasia type 2^8^, choroidal neovascularization and geographic atrophy secondary to AMD^9,10^, constitutes a surrogate outcome measure, but without the retest-variability of psychophysical testing. In these diseases, average errors (MAE) between predicted and measured point-wise sensitives of 3.66 dB to 4.64 dB could be achieved^8–10^. Such AI-based functional maps of the posterior pole, as shown in Fig. 2, greatly exceed the possibilities of psychophysical testing in terms of area coverage, spatial resolution, and repeatability^6^. This approach could also be applied to obtain an estimate of function in patients overchallenged with psychophysical testing or at clinical sites without dedicated FCP devices.

In terms of accuracy, MAE estimates for the inference of point-wise retinal sensitivity are in a similar range to the retest-reliability of FCP^20,21^, and similar to AI-based structure–function analyses in other retinal diseases^8–10,22^. Importantly, the predictions were overall unbiased. However, for loci with very low sensitivity (for which only few training examples were available), inferred sensitivity tended to overestimate function. The feature importance values also support the biological plausibility of the model. Specifically, ONL thickness, which can be interpreted as a surrogate of ONL cell count^23^, and the OS compartment thickness, which reflects the severity of SRF, constituted the most important features to estimate sensitivity at the same visit. Comparing scenario 1 to scenario 2 highlighted that the accuracy improves markedly with addition of patient-specific training data and inclusion of the patient identification number as explanatory variable. This highlights that patient-specific factors, which are not readily visible in SD-OCT data, influence sensitivity. This could include lenticular absorption or behavioral factors (“trigger-happy” patients). Thus, it appears reasonable to acquire some patient-specific retinal sensitivity data with a brief FCP test during a clinical trial and include these in the modeling process instead of fully relying on predictions from structure^9,10^.

Future retinal sensitivity was predicted with moderate accuracy (cross-validated R^2^ of 0.368 for the prediction of mean sensitivity). The 2 most important features to predict future retinal sensitivity were related to photoreceptor degeneration. Specifically, ONL thinning below 40 µm and loss/thinning of IS was associated with poor future retinal sensitivity. In addition, RPEDC thickening (i.e., presence of a pigment epithelial detachment) was also associated with poor future retinal sensitivity. Interestingly, received treatment was already the fourth most important predictor for future retinal sensitivity. A better functional outcome was predicted for patients that will undergo half-dose PDT, which matches the primary outcome of the complete PLACE trial cohort^4^. This highlights the relevance of adequate treatment for cCSC, which is PDT with reduced settings (even after adjusting for all structural factors that may affect retinal sensitivity). Other features such as the OS compartment thickness, which was important for the prediction of current function, showed little importance for the prediction of future sensitivity. This is plausible given that SRF tends to resolve over time (especially in treated eyes) and is therefore by itself not necessarily linked to future function^4^. In contrast, ONL thinning appears to be associated with both poor current and future retinal sensitivity. Of note, these AI-based results are in accordance with previously human expert-based analyses, which includes the prognostic value of photoreceptor integrity (Supplementary Figure S3 [ONL and IS thickness])^24^, and of diffuse atrophic RPE (Supplementary Figure S3 [reflected by the choroidal min. signal intensity])^25,26^.

This study has various limitations. Theoretically, selective testing of rod function in cCSC with scotopic FCP would reveal greater change in sensitivity upon resolution of SRF than mesopic testing^6,27,28^. By extension, this would also apply to “inferred scotopic sensitivity”^9,10^. However, scotopic FCP testing was not performed in the context of the PLACE trial, and therefore not available for training of the models. Regarding the generalizability of the models, the risk of optimization bias has been minimized by applying nested cross-validation to strictly separate the assessment of the model performance (outer cross-validation) from hyper-parameter tuning (embedded inner cross-validation)^15^. Nevertheless, the applicability domain of the models is limited to the cCSC patients that met in- and exclusion criteria of the PLACE trial. With larger data-sets, an end-to-end convolutional neural network (CNN) architecture could have been developed to infer directly function from the imaging data^8^. While this could (potentially) improve the prediction accuracies, the here presented 2-step approach (CNN based segmentation [feature extraction], followed by a machine-learning regression model) is advantageous in terms of interpretability.

In summary, this study demonstrates that morphology is closely correlated with retinal sensitivity in cCSC, using data from a prospective randomized controlled clinical trial. Inferred sensitivity as a surrogate of retinal function can be considered as a (secondary) clinical trial outcome measure, given the prediction accuracy as well as the biological plausibility of the models. However, large disease specific training sets and external data for validation constitute important prerequisites for application. This would allow to map function beyond the possibilities of psychophysical testing in terms of retinal coverage and spatial resolution. Moreover, estimation of the future outcome in cCSC may be helpful to inform and manage patients in routine clinical practice.

## Methods

### Clinical trial

The multicenter randomized controlled treatment PLACE trial (clinicaltrials.gov, NCT01797861) compared the anatomic and functional efficacy and safety of half-dose PDT versus HSML in patients with cCSC^4,12^. The protocol of the trial has been previously published^4,12^. In brief, CSC-related symptoms and/or imaging findings had to be present for at least 6 weeks for inclusion in the study. In addition, patients had to exhibit subfoveal SRF on SD-OCT, 1 or more regions of active leakage ('hot spots') on fluorescein angiography, and hyperfluorescent changes typical of cCSC on indocyanine green angiography. Patients were randomized to receive either half-dose PDT or HSML. At 6–8 weeks post-treatment, Evaluation Visit 1 was performed, during which anatomical (SRF on SD-OCT) and functional outcome (retinal sensitivity on microperimetry, best-corrected visual acuity, and outcome of a questionnaire on visual functioning) were assessed. Following treatment at baseline, the treatment was administered a second time at week 6–8 if necessary, i.e. when there was persistent SRF on SD-OCT. The final visit was at 7–8 months post-treatment and included the assessment of structural and functional treatment outcome measures. The presented analysis in this manuscript is based on the subset of patients enrolled at Leiden University Medical Center (Leiden, the Netherlands).

### Fundus-controlled perimetry testing and retinal imaging

FCP testing was performed using the MAIA device (Centervue SpA, Padova, Italy) at baseline, week 6–8 and at month 7–8 post-treatment. The test pattern was a radial pattern with a central stimulus and 3 rings at 1°, 3°, and 5° eccentricity from the fovea with 12 stimuli each (total of 37 stimuli). Testing was conducted with a mesopic background (background luminance 1.27 cd/m^2^) and a 4–2 staircase strategy. At all 3 visits, SD-OCT B-scans were acquired with a Heidelberg Spectralis device (Heidelberg Engineering, Heidelberg, Germany; 20° × 20°, 49 B-scans).

### Deep-learning-based SD-OCT segmentation

**Figure 3:**
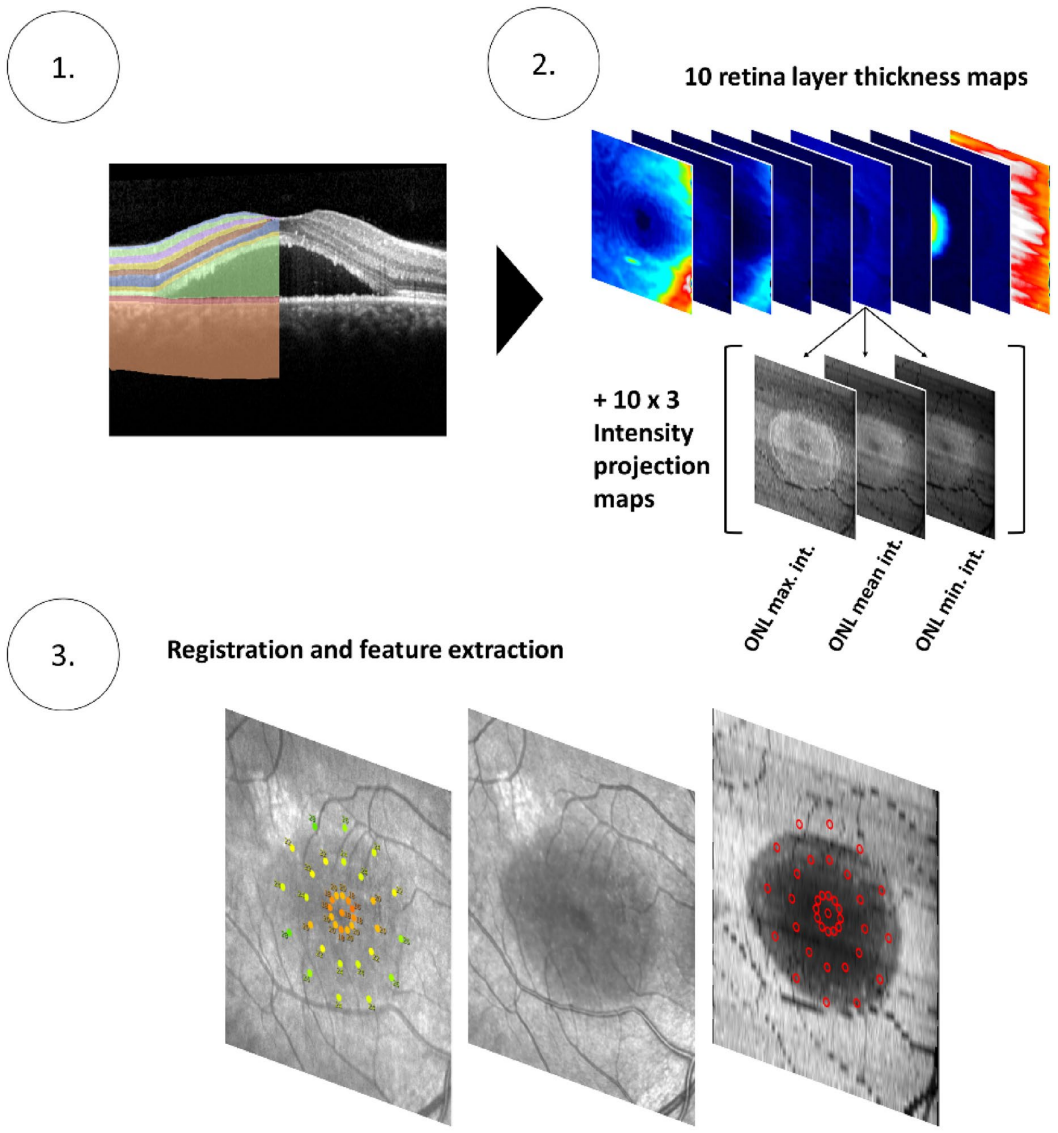
Image segmentation and feature extraction. The spectral domain-optical coherence tomography (SD-OCT) volumes were segmented using a custom deep-learning based pipeline (panel 1). Of note, subretinal fluid was counted toward the outer segment compartment. Subsequently (panel 2), thickness maps as well as 3 intensity projections per retinal layer were generated (total of 40 *en face* maps). The intensity projects depict the maximum, mean or minimum reflectivity within a given layer along each A-scan. Last (panel 3), the MAIA data was registered to the SD-OCT volume with the help of the co-acquired infrared reflectance image based on landmarks such as vascular bifurcations. This allowed to extract retinal layer thickness and reflectivity values corresponding precisely to the stimulus position and area.

The following SD-OCT layers were segmented using a custom, previously validated deep-learning-based pipeline (convolutional neural network architecture: DeepLabv3)^13^: retinal nerve fiber layer (RNFL), ganglion cell layer (GCL), inner plexiform layer (IPL), inner nuclear layer (INL), outer plexiform layer (OPL), ONL, IS, OS, RPEDC and choroid (CHO), as shown in Fig. 3. Of note, Henle’s fiber layer was counted toward the ONL^14^, SRF was counted toward the OS compartment, and the RPEDC included retinal pigment epithelium detachments. Next, retinal layer thickness maps and intensity projections were generated. For each layer, the intensity projections (3 per layer) depict the maximum, mean or minimum reflectivity along a given A-scan (Fig. 3).

### Extraction of imaging features

FCP data were then registered to SD-OCT data by using the co-acquired infrared reflectance image of the Spectralis device. Scale-invariant feature transform correspondences were extracted automatically for the MAIA infrared reflectance and Spectralis infrared reflectance image, which could then be applied for a subsequent affine transformation of the MAIA infrared reflectance image. Once the images were registered, retinal layer thickness and reflectivity values were extracted for each test point with a circular region of interest corresponding to the exact stimulus position and area (diameter of 0.43°).

### Predictive modeling

The prediction of retinal sensitivity for 3 clinically relevant scenarios was evaluated using the R packages *randomForest* and *caret*^15^. As learning algorithm, *random forest* regression was applied given the overall good performance in the context of collinearity (e.g., correlation of the retinal layer thicknesses). For all analyses, nested resampling was applied to assess the model accuracy (outer patient-wise leave-one-out cross-validation), while simultaneously optimizing the *random forest* parameter “mtry” (nested inner fivefold cross-validation).

Three clinically relevant scenarios were evaluated (graphically described in Supplementary Figure S1):

- For scenario 1 (accuracy of inferred sensitivity [same visit as SD-OCT] in absence of any patient-specific training data): Local retinal sensitivity constituted the dependent variable and the corresponding 40 retinal layer thickness/reflectivity values constituted the independent variables. The model was iteratively trained using data from *n-1* patients and tested on the data of the one remaining patient (outer patient-wise leave-one-out cross-validation).
- For scenario 2 (accuracy of inferred sensitivity [same visit as SD-OCT] with addition of the patient-specific baseline data to the training sets): In contrast to scenario 1, FCP and imaging data of the baseline visit of all patients was added to all training folds (and removed from the test folds). Further, the patient identification number was added through one-hot encoding to the predictor set to allow the regression model to learn patient-specific relationships that are otherwise poorly represented in SD-OCT data (e.g., lenticular opacification).
- For scenario 3 (prediction accuracy of sensitivity at the last visit, without any FCP data and solely based on one-time imaging at baseline for a specific patient): The 40 retinal layer thickness/reflectivity values of the baseline visit and treatment randomization (HSML vs. half-dose PDT) were considered as independent variables and point-wise retinal sensitivity at month 7–8 post-treatment as a dependent variable. Again, models were iteratively trained on n-1 patients and performance was assessed using the remaining patients (outer patient-wise leave-one-out cross-validation).

For all scenarios, the permutation importance [% IncMSE] was evaluated as measure of feature importance. The R package *forestFloor* was used to obtain feature contribution plots to visualize the underlying relationships^16^.

### Statistical analyses

All statistical analyses were performed using a mixed effects model to consider the hierarchical structure of the data (test point nested in eye as random effects term)^17^. The cross-validated MAE (based on a mixed effect model) between predicted and observed point-wise retinal sensitivity served for all 3 scenarios as measure of mode performance. In addition, the marginal R^2^ between cross-validated predictions and the observed value was computed, representing variance explained by the predictions^18^.

## Supplementary Information

Supplementary Figures.

## Data Availability

Original data will be shared by the corresponding author on reasonable request.
