# Supplementary material for: Estimation of current and post-treatment retinal function in chronic central serous chorioretinopathy using artificial intelligence
Source: Sci Rep. 2021 Oct 14;11:20446. doi: 10.1038/s41598-021-99977-4 (PMC8516921; doi:10.1038/s41598-021-99977-4)
Supplement: Supplementary file 1 — Supplementary Figures. [file 41598_2021_99977_MOESM1_ESM.docx]

**Supplementary Online Content**

**Estimation of Current and Post-Treatment Retinal Function in Chronic Central Serous Chorioretinopathy using Artificial Intelligence**

Maximilian Pfau*, Elon H.C. van Dijk*, Thomas J. van Rijssen, Steffen Schmitz-Valckenberg, Frank G. Holz, Monika Fleckenstein, Camiel J.F. Boon

|  | Page(s) |
| --- | --- |
| **Supplementary Figure S1. Prediction scenarios** | 2 |
| **Supplementary Figure S2. Improvement of accuracy through addition of patient-specific baseline data** | 3 |
| **Supplementary Figure S3. Prediction of the treatment potential** | 4 |

**Supplementary Figure S1. Prediction scenarios**

The figure shows the 3 prediction scenarios, which were evaluated in this study. In scenario 1, retinal sensitivity at baseline and its change over time at the both follow-up visits was inferred without any patient-specific fundus-controlled perimetry (FCP) data and solely based on imaging data, applying outer patient-wise leave-one-out cross-validation. For scenario 2, the patient identification number was added to the feature set and baseline data including FCP testing of all patients was added to the training folds in order to infer retinal sensitivity at both follow-up visits. This approach modelled the situation that a single FCP exam would have been done in a specific patient at baseline. These baseline patient-specific data would be then used to estimate retinal sensitivity at a follow-up visit when just additional retinal imaging would be carried out in this specific patient. In scenario 3, baseline spectral-domain optical coherence tomography (SD-OCT) features constituted the model input and retinal sensitivity at month 7-8 the output. Therefore, the model predicts the “treatment potential” of patients which is just assessed one-time at initiation of the intervention.


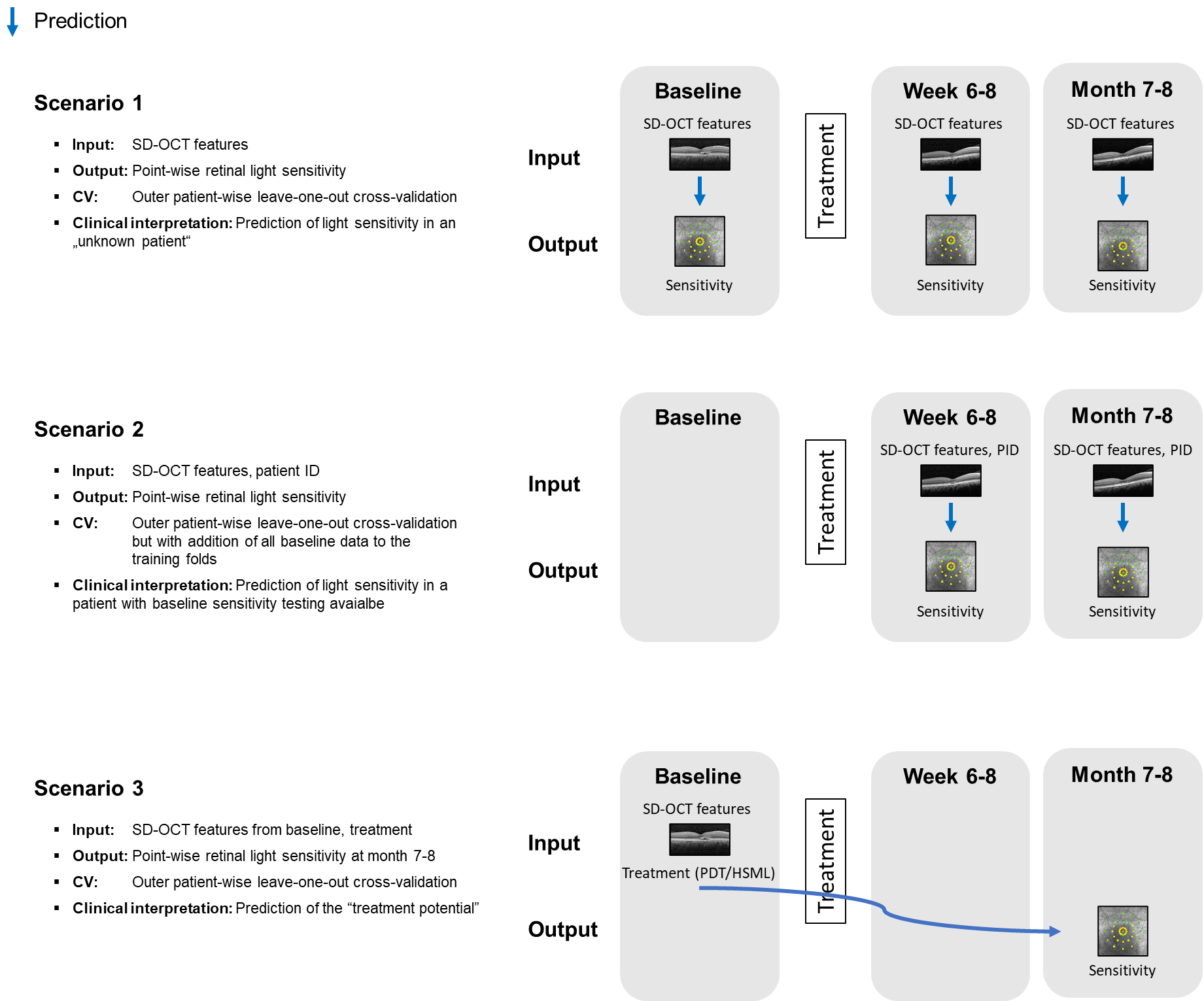


Abbreviation: Cross-Validation (CV)**Supplementary Figure S2. Improvement of accuracy through addition of patient-specific baseline data**

The upper row (A) shows the patient-wise mean absolute errors (MAE) between estimated and observed sensitivity (y-axis) for scenarios 1 and 2 (x-axis) and for each of the 3 visits (facets), respectively. As baseline data was added to the training data, no MAE for scenario 2 is shown for the baseline visit (left facet). The red dots indicate the average estimate for the MAE and the error bars indicate the 95% confidence intervals.

In the lower row, the Bland-Altman plots show the agreement between estimated and observed point-wise retinal sensitivity (pws) for scenario 1 (panel B) and scenario 2 (panel C). In contrast to the Bland-Altman plot in Figure 2, panel B only shows data for the week 6-8 post-treatment and month 7-8 post-treatment visit (scenario 1) to allow direct comparison to panel C (scenario 2). Note that agreement is much improved in scenario 2, particularly regarding sensitivity values at the lower end (dots on the left part of the panels).


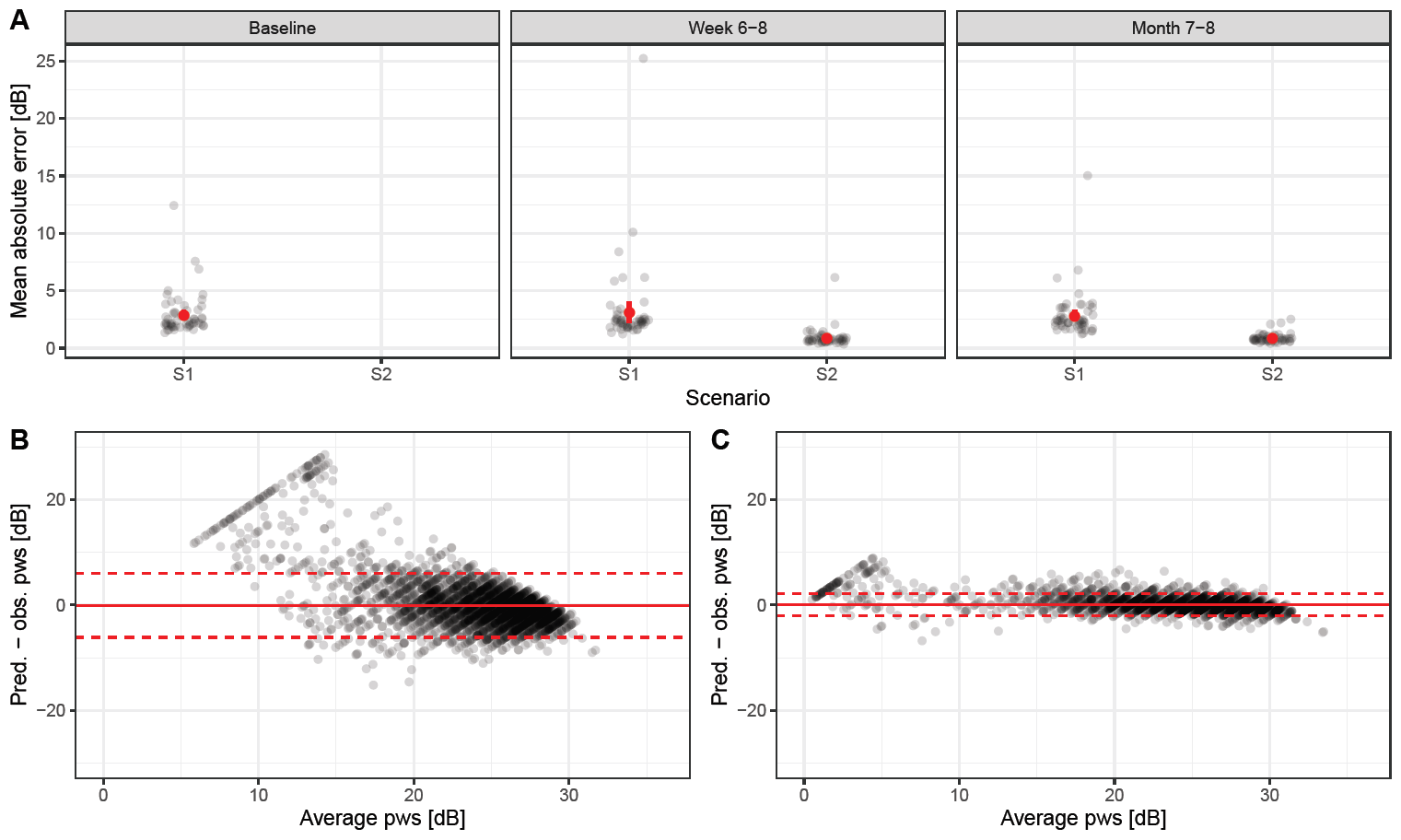


**Supplementary Figure S3. Prediction of the treatment potential**

Panel A shows the cross-validated predicted mean retinal sensitivity for month 7-8 post-treatment (x-axis) using the baseline structural data and the actually observed mean retinal sensitivity at month 7-8 post-treatment (y-axis).

Panel B shows the permutation feature importance in terms of the percentage increase in mean squared error (%IncMSE) for the 10 most important predictors. Each dot denotes the feature importance estimate for a given iteration of the outer cross-validation. The red vertical line indicates the median.

The panels C to F show the feature contribution plots for the 4 most important predictors. Notably, treatment with half-dose photodynamic therapy (PDT) results in a higher predicted retinal sensitivity at month 7-8 post-treatment as opposed to treatment with high-density subthreshold micropulse laser (HSML).


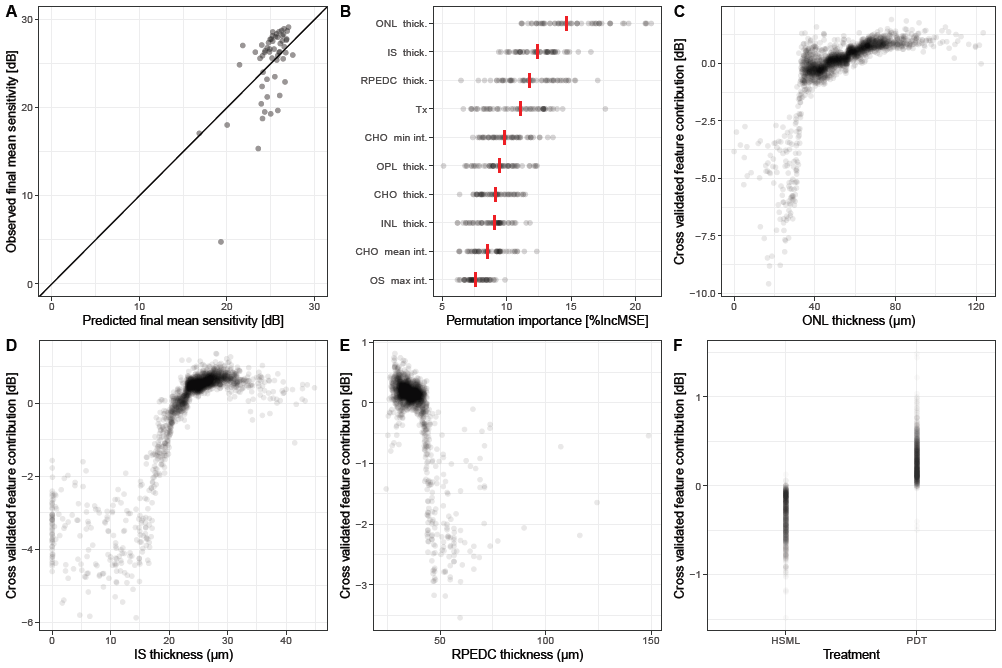


Abbreviations: prediction (pred.), observation (obs.), point-wise retinal sensitivity (pws), thickness (thick.), intensity (int.); Retinal layer: inner nuclear layer (INL), outer nuclear layer (ONL), outer segments (OS), inner segments (IS), retinal pigment epithelium-drusen complex (RPEDC), choroid (CHO)
